# Venetoclax and hypomethylating agents synergize to increase cell death and metabolic remodeling in acute B-lymphoblastic leukemia cells

**DOI:** 10.1016/j.molmet.2026.102402

**Published:** 2026-06-17

**Authors:** Patricia Maass, Sandra Lange, Felix Wittig, Nares Trakooljul, Frieder Hadlich, Anett Sekora, Christian Schmidt, Hugo Murua Escobar, Klaus Wimmers, Burkhard Hinz, Christian Junghanss, Anna Richter

**Affiliations:** 1 Department of Internal Medicine, Clinic for Hematology, Hemostasis, Oncology, Stem Cell Therapy and Palliative Medicine, Rostock University Medical Center, Ernst-Heydemann-Str. 6, 18057 Rostock, Germany; 2 Institute of Pharmacology and Toxicology, Rostock University Medical Center, Schillingallee 70, 18057 Rostock, Germany; 3 Research Institute for Farm Animal Biology, Wilhelm-Stahl-Allee 2, 18196 Dummerstorf, Germany; 4 Institute of Medical Genetics, Rostock University Medical Center, Ernst-Heydemann-Str. 8, 18057 Rostock, Germany

**Keywords:** Acute lymphoblastic leukemia, Venetoclax, Azacytidine, Decitabine, Hypomethylating agents, Metabolism, Single-cell sequencing

## Abstract

**Introduction:**

Overexpression of anti-apoptotic protein BCL-2 and hypermethylation are hallmarks of acute lymphoblastic leukemia (ALL) and can be pharmacologically addressed by venetoclax (VEN) and hypomethylating agents (HMA) such as azacytidine (AZA) or decitabine (DEC). Combined VEN and HMA application was recently successfully implemented into the clinical treatment regimen of acute myeloid leukemia but has so far not been investigated in ALL.

**Methods:**

We therefore analyzed the anti-leukemic potential of VEN + HMA in four ALL cell lines and identified potential modes of synergy to overcome mono-drug-induced resistance using proliferation, metabolism, methylation and apoptotic protein expression assays. Single cell RNAseq of a VEN-treated PDX model was used to gain deeper insights into metabolic reprograming.

**Results:**

All substances influenced proliferation and induced apoptosis in a subset of cell lines. Combined VEN and HMA application resulted in significantly reduced metabolic activity. In contrast, no synergistic effects were observed regarding the BCL-2 protein and methyltransferase expression or global methylation. Single cell RNAseq revealed that VEN interferes with both main energy supply routes, oxidative phosphorylation as well as glycolysis, to impede the cells’ metabolism and mitochondrial activity. The addition of HMA, especially DEC, increased anti-metabolic effects, leading to a strong reduction of respiration, ATP production and proton leakage. AZA-induced metabolic suppression and overall anti-leukemic activity alone and in combination with VEN was generally weaker compared to DEC.

**Conclusion:**

Altogether, we herein demonstrate that combined VEN and HMA application acts synergistically and significantly reduces the leukemic burden in ALL cell lines via impairment of tumor cell metabolism and mitochondrial function.

## Highlights

• VEN + HMA reduces proliferation and metabolism in ALL cell lines.
• Synergy is evoked via dual metabolic targeting.
• Combination blocks oxidative phosphorylation and glycolysis.
• DEC combinations are superior to AZA.

## 1 Introduction

Acute B-lymphoblastic leukemia (B-ALL) is an aggressive malignancy affecting both, infants and young children as well as adults. While pediatric B-ALL patients can often be cured, the prognosis declines with age and results in a five-year overall survival rate of only 25 % in patients older than 50 years [1]. B-ALL is a cytogenetically heterogeneous disease, resulting in increasing incorporation of targeted therapies and personalized medicine. Among others, the BCL-2 inhibitor venetoclax (VEN) demonstrated good preclinical activity in B-ALL models [[2], [3], [4]] and promising early clinical results [5,6]. However, both phase I/II studies observed high rates of secondary resistance.

Similarly, secondary VEN resistance frequently occurs in acute myeloid leukemia (AML) as well as chronic lymphoblastic leukemia (CLL), where VEN is part of the standard therapeutic regime [7,8]. Combinatory approaches are therefore required to increase therapeutic efficacy and delay the occurrence of resistance. In AML, VEN is applied together with hypomethylating agents (HMA) like azacytidine (AZA) or decitabine (DEC), resulting in improved therapeutic outcomes [9,10]. Comparable to VEN, HMA have previously been investigated in B-ALL, with promising preclinical and early clinical findings [[11], [12], [13], [14], [15], [16]]. Apart from one preclinical report, combined VEN and HMA application has not been investigated for B-ALL so far. Cheung et al. showed increased survival rates in infant ALL xenograft models following VEN and AZA treatment, underlining the therapeutic potential of this approach [17]. However, mechanistic insights investigating the molecular background of this synergy are yet to be conducted, and combinatory approaches using DEC and including adult B-ALL models are needed to better understand the underlying mechanisms. We have recently shown that DEC not only degrades its known target DNA methyltransferase 1, but also leukemic driver KMT2A [18]. Fusion transcripts of KMT2A are known to induce BCL2 expression [4], suggesting that combined DEC and VEN treatment might synergize in this aspect.

In light of the generally high BCL-2 expression and hypermethylated status of B-ALL, combined VEN and HMA application might offer a beneficial addition to the therapeutic options of B-ALL patients. In this study, we investigated the effects of VEN and HMA treatment on proliferation, cell death and metabolic processes in B-ALL cell lines, demonstrating that synergistic anti-leukemic mechanisms were mainly attributed to metabolic reprogramming and impaired ATP production.

## 2 Materials and methods

### 2.1 Cell culture and PBMC isolation

Human B—ALL cell lines SEM (*KMT2A::AFF1*-rearranged), RS4;11 (*KMT2A::AFF1*-rearranged), REH (*ETV6::RUNX1*-rearranged) and NALM-6 (*ETV6::PDGFRB*-rearranged) were purchased from DSMZ (Braunschweig, Germany) and maintained at 37 °C and 5 % CO_2_ in IMDM medium (SEM), Alpha MEM medium (RS4;11) or RPMI medium (REH, NALM-6) supplemented with 10 % heat-inactivated fetal calf serum (FCS) and 100 μg/ml penicillin/streptomycin (all PAN—Biotech, Aidenbach, Germany). Medium was changed twice weekly and cells were seeded at a density of 3.3 × 10^5^ cells per ml for further cultivation. Cells were regularly checked for authenticity (cell surface flow cytometry) and mycoplasma contamination. Peripheral blood of three healthy donors was collected and peripheral blood mononuclear cells (PBMC) were isolated using PANcoll (PAN-Biotech) and density gradient centrifugation.

### 2.2 Drug exposure

For exposure experiments, cells were seeded at a density of 3.3 × 10^5^ cells per ml. Venetoclax (VEN), Azacytidine (AZA) and Decitabine (DEC) were all purchased from MedChemExpress (Monmouth Junction, NJ, USA) and dissolved in DMSO. For combination experiments, drugs were either applied simultaneously (drug1+drug2), or sequentially, with one drug given from the beginning and the other added after 24 h (drug1>drug2). The final solvent concentration per compound for exposure experiments did not exceed 0.1 % (v/v) DMSO for both, mono and combination treatment experiments. Incubation times and drug concentrations are given in the respective figure legends.

### 2.3 Viability and vitality assays

Cell proliferation was assessed by either CyQUANT Direct Cell Proliferation Assay (ThermoFisher Scientific, Waltham, MA, USA) according to the manufacturer's guidelines for cross-titration studies or trypan blue dye exclusion and microscopic quantification of viable cells. Metabolic activity was evaluated by WST—1 assay (Roche, Mannheim, Germany) in three technical replicates. Synergy maps of cross-titration experiments were generated using SynergyFinder 3.0 [19] based on calculations using the zero interaction potency model. For calculation of synergistic potential following simultaneous or sequential application, the Bliss independence model was used [20].

Drug concentrations for combination studies were selected based on previous dose finding studies [2,11]. To evaluate toxicity in healthy non-neoplastic blood cells, triplicates of 5 × 10^4^ PBMC per well were seeded into a 96-well plate and incubated with inhibitors for 24 h. Afterwards, 50 μl Calcein AM working solution (Sigma–Aldrich, St. Louis, MO, USA; final concentration 0.5 μM) per well were added and incubated for 30 min before detection using the Tecan plate reader (Tecan, Männedorf, Switzerland; excitation 485 nm, emission 535 nm). To analyze potential hemolysis, 20 μl full blood was distributed into a 96-well plate in technical triplicates and incubated with inhibitors, DMSO (negative control) or 2 % Triton™ X-100 (Merck, Darmstadt, Germany, positive control) for 120 min. Absorption of the cell-free supernatant was determined at 540 nm (reference 690 nm).

For analysis of apoptosis induction, cells were incubated with drugs and combinations, resuspended in binding buffer (BD, Heidelberg, Germany) and subsequently stained with annexin V—FITC (BD) for 15 min. Propidium iodide (PI, 0.6 μg/ml) was added immediately prior to flow cytometric analysis using the FACSVerse (BD) with FACSuite software (BD). Annexin V^+^ PI^−^ cells were considered as early apoptotic and annexin V^+^ PI^+^ cells as late apoptotic or necrotic. Annexin V^−^/PI^−^ cells were designated as vital. To investigate cell cycle phases, cells were incubated with drugs before fixation in ice-cold 70 % ethanol for 24 h at −20 °C. Cells were incubated with 1 mg/ml ribonuclease A (Sigma–Aldrich) at 37 °C for 45 min before staining with 50 μg/ml PI and analysis by flow cytometry (FACSVerse (BD) with FACSuite software (BD)).

### 2.4 Assessment of morphology

Cytospins were prepared by spinning 5-10 × 10^4^ cells onto microscopic slides using a Cytospin 3 centrifuge (Shandon, Frankfurt/Main, Germany, 10 min, 700 rpm). Slides were air-dried and for Pappenheim staining first incubated in May-Grünwald solution (Merck), then washed in buffer solution according to Weise (pH 7.2, Sigma–Aldrich), stained in Giemsa solution (Merck) for 20 min, and finally washed with buffer according to Weise followed by A. dest. Images were captured using a 100—fold objective.

### 2.5 Gene expression analysis

RNA isolation was carried out using the RNeasy Mini Kit (Qiagen, Hilden, Germany) and followed by cDNA synthesis facilitating the PrimeScript Reagent Kit (Takara Bio Europe, Saint-Germain-en-Laye, France) according to the manufacturer's guidelines. Gene expression was measured in technical triplicates in a ViiA7 Real Time PCR system (Applied Biosystems, Foster City, USA) using the SensiFAST™ Probe Lo-ROX kit (Meridian Bioscience, Cincinnati, OH, USA), 0.5 μM primers (Table S1), 0.1 μM probe (Table S1) and 20 ng cDNA in a final volume of 13 μl. For *BCL2*, a commercial gene expression assay was used (Hs04986394_s1, ThemoFisher Scientific, Dreieich, Germany). The PCR consisted of 2 min 50 °C and 10 min 95 °C initial denaturation followed by 45 cycles of 15 s denaturation at 95 °C and 1 min annealing/elongation at 60 °C. Gene expression was normalized to sample-matching *GAPDH* housekeeping gene values and the ΔΔCt was calculated for comparison of controls and treated samples.

### 2.6 Global methylation analysis

Genomic DNA was extracted using the NucleoSpin® Tissue Kit (Macherey–Nagel, Dueren, Germany) and AllPrep DNA/RNA Kit (Qiagen) for in vitro and in vivo samples, respectively. Bisulfite conversion was carried out according to the manufacturer's guidelines using the EpiTect Bisulfite Kit (Qiagen). LINE-1 methylation-specific qPCR was carried out on a ViiA7 system (ThermoFisher Scientific) in a final volume of 20 μl containing 20 ng of bisulfite-treated DNA, Quantitect SYBR Green PCR Master Mix (Qiagen) and 0.25 μM primers specific for either methylated or unmethylated DNA (Table S2) as follows: initial denaturation (15 min, 95 °C) followed by 45 cycles of denaturation (15 s, 94 °C), annealing (30 s, 55 °C) and elongation (30 s, 72 °C).

### 2.7 BCL-2 family protein expression analysis by intracellular flow cytometry

For quantification of BCL-2, p-BCL-2, MCL-1, BCL-xL and BAX, cells were harvested from in vitro experiments or murine spleens and washed in cold PBS, fixed in methanol-free 4 % formaldehyde (Polysciences, Warrington, PA, USA) for 15 min and washed twice in PBS before permeabilization in ice-cold 90 % methanol for 30 min. After two steps of PBS washing, cells were blocked in antibody dilution buffer for 10 min and incubated with the antibodies listed in Table S3 for 35 min at room temperature. Cells were again washed twice in antibody dilution buffer and analyzed using the FACSLyric™ device (BD) with FACSuite™ software (BD; version 1.0.6.5230).

### 2.8 Mitochondrial stress analysis

To determine the oxygen consumption rate (OCR), extracellular acidification rate (ECAR) as well as mitochondrial stress parameters, the Seahorse XF Cell Mito Stress Test was performed using the Seahorse XFe 24 Flux analyzer (both Agilent Technologies, Santa Clara, CA, USA). SEM cells were incubated with DMSO, 10 nM VEN, 1 μM AZA, 100 nM DEC or VEN/HMA combinations for 72 h. Prior to the assay the cartridge was hydrated overnight with 1 ml Seahorse Calibrant (Agilent Technologies) per well. Also, the Seahorse 24-well plate was coated with 0.1 mg/ml poly-d-lysine (Sigma–Aldrich) in DPBS for at least 1 h before the seeding to promote temporary adherence of the suspension cells. A total number of 6 × 10^5^ SEM cells per well were then transferred into the Seahorse plate in technical triplicates to the Seahorse plate in Seahorse XF base medium (Agilent Technologies) supplemented with 10 mM glucose, 2 mM glutamine and 1 mM pyruvate (referred to as Seahorse full medium). Seahorse full medium served as blank controls. The plate was then centrifuged at 200×*g* for 3 min to immobilize the cells and subsequently incubated for 2 h to equilibrate in a non-CO_2_ incubator at 37 °C before OCR and ECAR measurement. For the Mito Stress Test, the inhibitors oligomycin (final concentration 1.5 μM), FCCP (2–4 μM) and rotenone & antimycin A (0.5 μM) were prepared in Seahorse full medium and loaded into the ports of the cartridge.

### 2.9 Lipid peroxidation assay

The lipid peroxidation assay uses a ratiometric peroxidation sensor that changes its fluorescence from red to green when detecting lipid peroxidation. For this assay, 5 × 10^5^ cells were incubated with DMSO, VEN or HMAs alone or in simultaneous combination for 72 h. For each sample, 90 μl cell suspension was transferred into a 96-well plate and 10 μl of lipid peroxidation sensor (Abcam, Milton, UK) were added and incubated for 30 min at 37 °C and 5 % CO_2_. Cells were then transferred to FACS tubes and washed three times with 2 ml PBS and resuspended in 500 μl PBS. The PE and FITC intensities were measured with the FACSVerse (BD) and FACSuite™ software (BD). PE/FITC ratio calculation and normalization to DMSO controls was performed using FlowJo software (version 10.10, BD).

### 2.10 Flowcytometric GPX4 analysis

Cells were treated with DMSO, VEN, HMAs or combination for 72 h before harvest and PBS washing. Cells were fixed for 15 min in 300 μl 4 % formaldehyde (Polysciences) and subsequently washed in 3 ml PBS. The pellet was dissolved in 100 μl ice cold PBS and permeabilized by adding 900 μl ice cold methanol for 10–20 min before addition and washing in 2 ml PBS. Cells were then washed in 2 ml PBS/1 % FCS (PAN-Biotech) and blocked for 10 min before incubation with 0.8 μg GPX4 antibody (Proteintech, Manchester, UK; Table S3) for 40 min at room temperature. Following two washing steps in 2 ml PBS/1 % FCS, cells were resuspended and analyzed using FACSVerse (BD) and FACSuite™ software (BD).

### 2.11 FerroOrange assay

Cells were treated with DMSO, VEN, HMAs or combination for 72 h before harvesting, two washing steps in PBS and determination of absolute cell count by trypan blue staining. 5 × 10^5^ cells in 50 μl per well were seeded in technical duplicates and 50 μl of 2 μM FerroOrange working solution (CellSignaling, Danvers, MA, USA) were added to each well and incubated at 37 °C for 30 min. The plate was then measured using a Tecan plate reader device (540 nm/585 nm).

### 2.12 In vivo application of venetoclax

Animal experiments were approved by the review board of the federal state Mecklenburg-Vorpommern, Germany (LALLF MV/7221.3-1.1-063/20) and performed in accordance to the Declaration of Helsinki and the local ethical standards of the Rostock University Medical Center (A2018-0122). The donor of the ALL primary cells gave informed consent. Three to four months old male and female NOD.Cg-*Prkdc*^*scid*^*Il2rg*^*tm1Wjl*^/Szj (NSG) mice were bred and housed in the accredited laboratory animal Core Facility of the Rostock University Medical Center with access to water and standard chow *ad libitum*. Experiments were carried out in a laboratory setting and no intervention was performed within the animal housing and breeding rooms. Tumor cell injection, monitoring of blast counts using peripheral blood flow cytometry (CD45^+^/CD19^+^) and tumor cell isolation were performed as previously described [11,[21], [22], [23]]. Mice were monitored and weighed daily. Three mice each were engrafted with primary cells previously expanded in NSG mice and continuously treated with either VEN or vehicle on three days per week starting seven days after tumor cell injection (i.v., 2.5 × 10^6^ cells per animal). Randomization was performed based on sex, age and weight. Study groups were not blinded to the investigators. VEN was dissolved in 60 % Phosal 50 PG (Lipoid, Ludwigshafen, Germany), 30 % PEG400 (Carl Roth, Karlsruhe, Germany) and 10 % ethanol and applied via oral gavage. During the first week of treatment, VEN doses were increased according to the clinically applied protocol, starting at 20 mg/kg body weight, and raised to the maximum dose of 100 mg/kg body weight at day 11. Mice were euthanized by narcotization (75 mg/kg ketamine, 5 mg/kg xylazine) followed by cervical dislocation when blast frequencies reached ≥30 % in blood. For single cell RNAseq, one representative control and one representative VEN-treated mouse were selected.

### 2.13 Single cell transcriptomics

Tumor cells were isolated from spleens of PDX animals as described before [23] and washed and diluted to 1 × 10^6^ cells per ml in PBS/0.04 % BSA. The Single Cell 3′ Gene Expression libraries were constructed using the Chromium Next GEM single Cell 3′ Reagent Kits v3.1, dual index (10x Genomics, Pleasanton, CA, USA) according to the manufacturer's guidelines. Approximately 10,000 cells were loaded onto a chip to generate Gel Beads-in-emulsion (GEMs) reactions using the Chromium Single Cell Controller version 4.0. cDNA samples and final libraries were quality-checked using a Agilent Bioanalyzer High Sensitivity Chip. The libraries were normalized to a final concentration of 650 pM and paired-end sequenced (Read1 for 28 cycles, i7-Index for 10 cycles, i5-Index for 10 cycles and Read2 for 90 cycles) on the NextSeq 2000 system (Illumina) at the NGS facility of the Research Institute for Farm Animal Biology (FBN), Germany.

Raw reads (fastq) were generated and demultiplexed using dragen bcl convert v3.10.11. Data were processed using nf-core/scrnaseq pipeline [24] and Cell Ranger v7.1.0 was used for alignment of the data to the GRCh38 human reference genome (Ensembl, version 109) and generation of count matrix. QC matrices and cell selection and filtration were carried out using Seurat [25]. Data were further processed using Azimuth and the Loupe Browser software (version 8.1, 10X Genomics). Enrichment analyses were conducted using Enrichr pipeline [[26], [27], [28]]. Raw data can be retrieved from the European Bioinformatics Institute's (EBI) platform BioStudies (accession number E-MTAB-14805).

### 2.14 Statistical analyses

All values are expressed as mean ± standard deviation (SD). Gaussian normality distribution was tested in all cases, determining the following parametric or non-parametric with post hoc test. The exact test is indicated in the respective figure legends. Kaplan–Meier curves and respective statistics were applied to estimate survival benefits. Statistical analyses were performed using GraphPad PRISM software (version 8). Statistical significance was defined as ∗ p < 0.05, ∗∗p < 0.01 and ∗∗∗p < 0.001.

## 3 Results

### 3.1 VEN and HMA influence proliferation, methylation and pathway-specific expression of B-ALL cell lines

**Figure S1:**
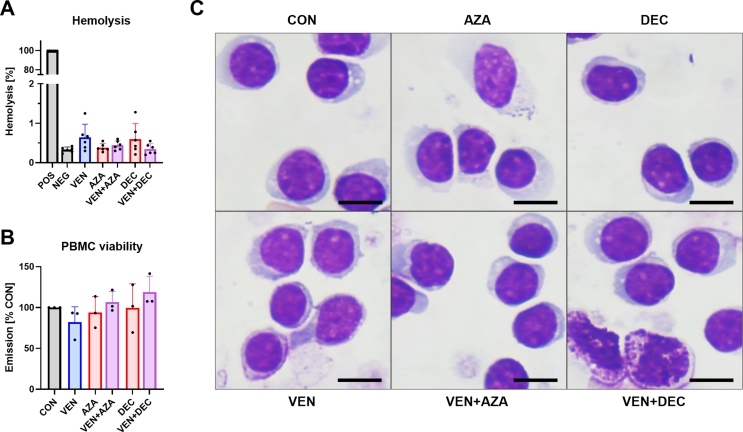
Effect of VEN and HMA on healthy blood cells. **A** In vitro analysis of hemolysis following 2 h incubation with highest VEN (50 nM) and HMA (1 μM) concentrations used in other assays, positive control (POS; Triton™ X-100) or negative control (NEG; DMSO). Analysis of full blood of six healthy voluntary donors. Mean ± SD, repeated measures one-way ANOVA and post-hoc Tukey's multiple comparisons test. ∗ p < 0.05 vs NEG. **B** Calcein AM-based in vitro PBMC viability assay of three healthy voluntary donors after 48 h incubation with CON, VEN (50 nM), HMA (1 μM) or both. Mean ± SD, Kruskal–Wallis test and post-hoc Dunn's multiple comparisons test. ∗ p < 0.05 vs CON. **C** Representative images of Pappenheim-stained cytospin preparations of healthy donor PBMCs treated with vehicle, VEN, HMA or combination. Scale bar, 20 μm.

Studying the influence of combined VEN and HMA application, we first investigated the effect of the drugs on healthy non-neoplastic blood cells using the maximum concentrations used for subsequent experiments in B-ALL cell lines. VEN, AZA and DEC did not significantly interfere with erythrocyte integrity (Fig. S1A) or PBMC viability (Fig. S1B) and did not alter PBMC morphology either alone or in combination (Fig. S1C), demonstrating that the selected drug doses are within the therapeutic window and suitable to investigate blast-specific effects.

**Figure 1:**
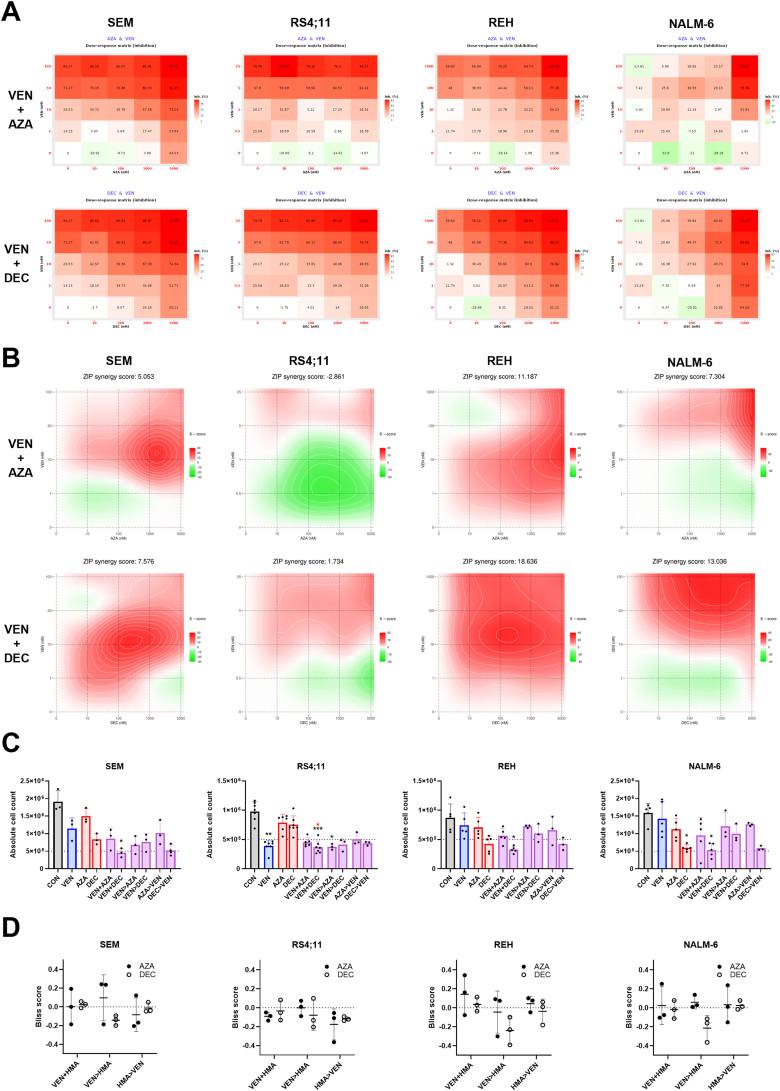
**Influence of combined VEN and HMA application on proliferation of ALL cells. A** Dose–response matrix of cross-titration experiments of combined VEN and HMA application in B-ALL cell lines SEM, RS4;11, REH and NALM-6 using increasing concentrations of VEN, AZA (upper panel) and DEC (lower panel). Red color indicates high inhibitory capacity while while or green shades suggests therapeutic resistance. **B** Synergy maps were generated using the SynergyFinder tool [19] based on the zero interaction potency (ZIP) model. Red color indicates synergistic potential while green color suggests antagonism. **C,D** For simultaneous treatment (VEN + HMA), cells were seeded and incubated with both inhibitors for 72 h. For sequential application (VEN > HMA or HMA > VEN), cells were seeded and immediately incubated with the first drug. After 24 h, the second drug was added for a total cultivation period of 72 h. **C** Trypan blue staining and subsequent microscopic quantification of vital cells following incubation with VEN (10 nM), HMA (AZA, 1 μM; DEC, 100 nM) or combined for 72 h. Each dot represents an individual biological replicate and the dotted line indicates the initially seeded cell number of 5 × 10^5^. Mean ± SD, repeated measures one-way ANOVA with post-hoc Dunnett's multiple comparisons test. ∗p < 0.05, ∗p < 0.01, ∗∗∗p < 0.001 vs CON (black), VEN (blue) or HMA (red). **D** Calculation of synergistic anti-proliferative activity according to the Bliss independence model based on the data presented in Figure 1C. Positive bliss scores indicate synergy while negative scores predict antagonism. Each dot represents an individual biological replicate.

To characterize the effect of combined VEN and HMA incubation on B-ALL cell proliferation, we first performed an in vitro screening using cross-titration in the four cell lines SEM, RS4;11, REH and NALM-6 (Figure 1A). Of note, NALM-6 cells are intrinsically resistant to VEN while none of the cell lines responded to AZA in physiologically achievable concentrations. In contrast to healthy non-neoplastic blood cells, the combinations enhanced the anti-proliferative effects of the mono substances. Calculating synergistic potential of the combined treatment revealed mainly additive effects (ZIP synergy scores −10 to 10) across wide ranges of cross-titration dosages (Figure 1B). Effects were stronger in samples incubated with DEC compared to AZA. Synergism (ZIP >10) was observed in VEN + DEC-incubated REH and NALM-6 cells as well as in REH cells following VEN + AZA application. However, those combinations disclosed highest synergistic potential using HMA concentrations that are well above physiological concentrations; we therefore conclude that simultaneous treatment with VEN and HMA does not induce significant and synergistic impairment of leukemic cell proliferation.

Also, the order of inhibitor application did not influence leukemia cell proliferation in great detail (Figure 1C). Calculation of the synergistic anti-proliferative potential revealed that combinations including AZA tended to be more effective when VEN was applied first, while the opposite or simultaneous incubation was best for DEC (Figure 1D).

**Figure S2:**
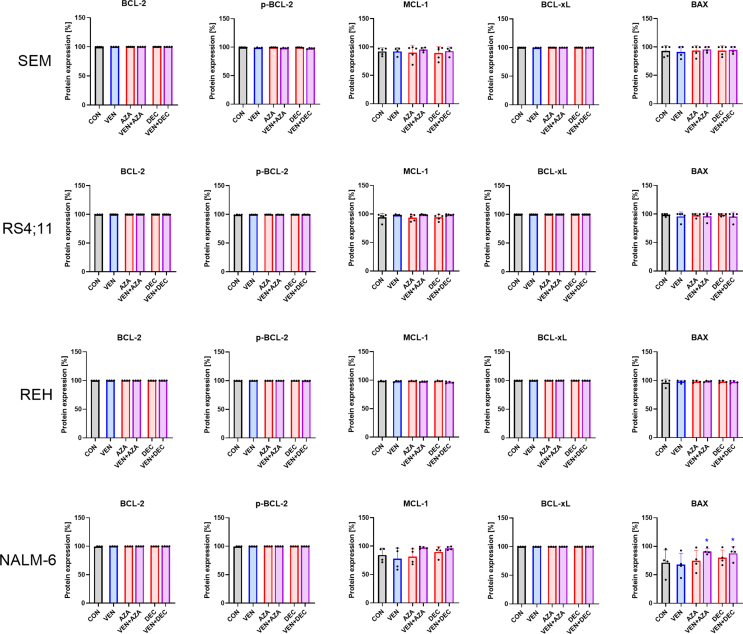
Effect of single and combined VEN and HMA treatment on absolute BCL-2 family protein expression. Flow cytometry to determine protein expression in B-ALL cell lines SEM, RS4; 11, REH and NALM-6. Each dot represents an individual biological replicate. Mean ± SD, Friedman test. ∗p < 0.05 vs CON.

**Figure S3:**
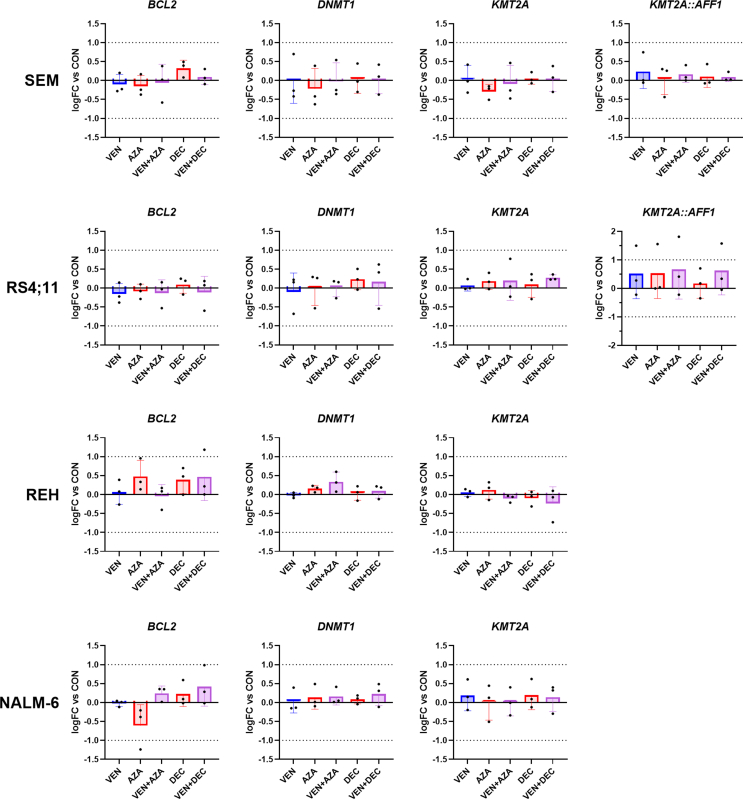
Influence of mono and combined application of VEN and HMA on gene expression profiles of B-ALL cell lines SEM, RS4; 11, REH and NALM-6. Inhibitors (VEN: SEM; 10 nM, RS4; 11, REH, NALM-6, all 2.5 nM; AZA, all 1 μM; DEC, all 100 nM) were applied for 24 h. Gene expression changes following inhibitor incubation compared to DMSO control. The dotted lines indicate a biologically relevant log-fold change (logFC) greater 1 or smaller −1. Each dot represents an individual biological replicate. Mean ± SD, Friedman and post-hoc Dunn's multiple comparisons test. ∗ p < 0.05 vs CON.

Further investigating known mechanisms of VEN and HMA action, we first analyzed if addition of AZA or DEC to BCL-2 inhibitor VEN interfered with protein expression of the BCL-2 pathway members (Fig. S2). Intracellular flow cytometric analysis demonstrated no significant mediation of BCL-2, p-BCL-2, MCL-1, BCL-xL and BAX protein expression following combined VEN and HMA treatment compared to mono application. In SEM cells, VEN and VEN-containing combinations resulted in slight downregulation of p-BCL-2, while mildly increased MCL-1 and BAX expression was observed in those samples in RS4;11 and NALM-6 cells, respectively. Matching protein expression data, gene expression of *BCL2* was also not synergistically altered in VEN, HMA or combination samples of any of the four cell lines investigated (Fig. S3).

**Figure S4:**
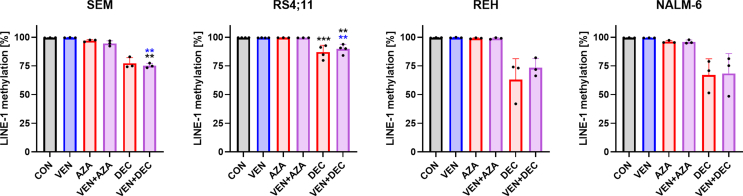
Influence of mono and combined application of VEN and HMA on absolute global methylation of B-ALL cell lines SEM, RS4; 11, REH and NALM-6. Inhibitors (VEN: SEM; 10 nM, RS4; 11, REH, NALM-6, all 2.5 nM; AZA, all 1 μM; DEC, all 100 nM) were applied for 72 h. Determination of absolute global methylation frequency using methylation-specific LINE-1 qPCR. Each dot represents an individual biological replicate. Mean ± SD, repeated measures one-way ANOVA with post-hoc Tukey's multiple comarisons test. ∗p < 0.05, ∗∗p < 0.01, ∗∗∗p < 0.001 vs CON (black), VEN (blue) or HMA (red).

DNA methylation is mediated via DNA methyltransferase 1 (DNMT1), which can be inhibited by HMA treatment. Neither HMA single application nor the combination with VEN altered *DNMT1* gene expression profiles (Fig. S3). The methyltransferase KMT2A, which is involved in leukemic progression, as well as the fusion transcript *KMT2A::AFF1* found in SEM and RS4;11 cells were also not influenced by any treatment on transcriptional level (Fig. S3). With AZA and DEC being demethylating agents and acting via DNMT1 degradation, we also analyzed the LINE-1 retrotransposon as a marker for global methylation (Fig. S4). DEC induced demethylation in all cell lines, and the addition of VEN did not increase the effect. In contrast, physiologically achievable concentrations of AZA did not result in profound changes in global methylation with only slightly decreased LINE-1 values in SEM and NALM-6 cells.

### 3.2 HMA accelerate VEN-induced cell death

**Figure 2:**
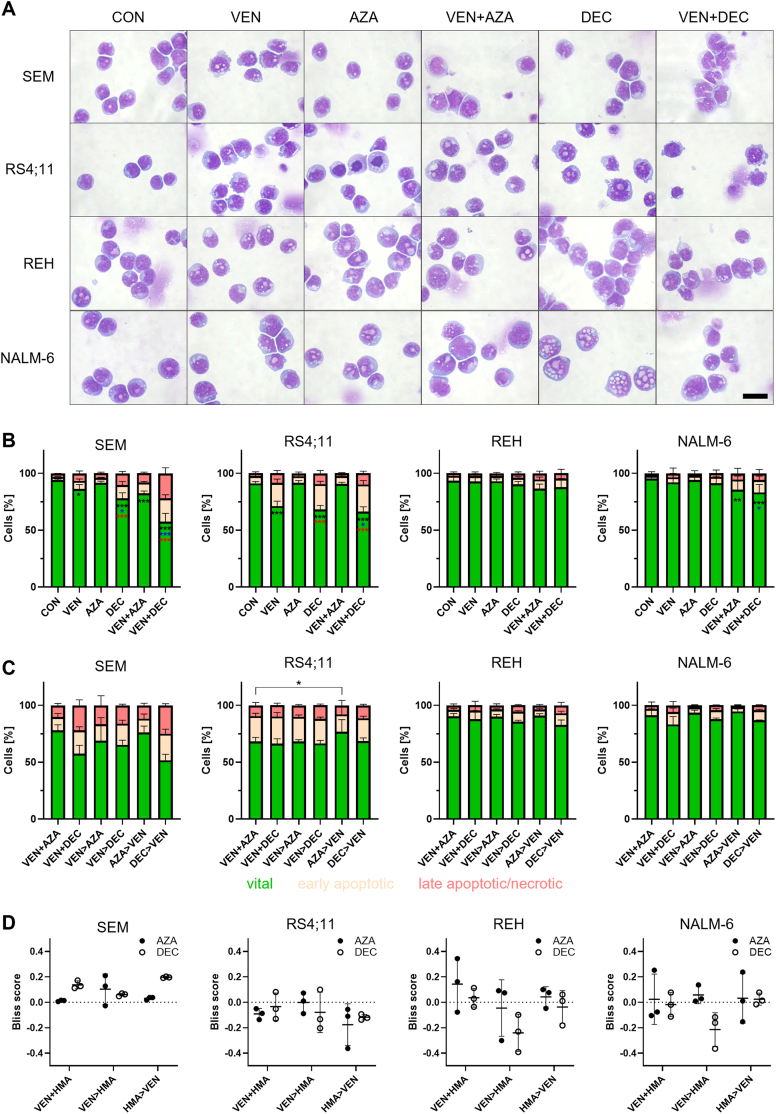
**Influence of VEN and HMA on cell death mechanisms in B-ALL cell lines SEM, RS4;11, REH and NALM-6.** For simultaneous treatment (VEN + HMA), cells were seeded and incubated with both inhibitors for 72 h. For sequential application (VEN > HMA or HMA > VEN), cells were seeded and immediately incubated with the first drug. After 24 h, the second drug was added for a total cultivation period of 72 h. VEN: SEM; 10 nM, RS4;11, REH, NALM-6, all 2.5 nM; AZA, all 1 μM; DEC, all 100 nM **A** Representative images of three independent biological replicates of Pappenheim-stained cytospin preparations. Scale bar, 50 μm. **B,C** Apoptosis analysis following incubation with inhibitors alone (**B**), simultaneously (**B,C**) or sequentially (**C**) combined for 72 h. Early apoptotic, annexin V+/PI-; late apoptotic/necrotic, annexin V+/PI+. N = 3, mean ± SD, 2-way ANOVA with post-hoc Tukey's multiple comparisons test. ∗p < 0.05, ∗∗p < 0.01, ∗∗∗p < 0.001 vs CON (black), VEN (blue) or HMA (red). **D** Calculation of synergistic apoptosis induction according to the Bliss independence model based on the data presented in Figure 2B,C. Positive bliss scores indicate synergy while negative scores predict antagonism. Each dot represents an individual biological replicate.

We next determined the effect of VEN and HMA application on the cells’ morphology (Figure 2A). All inhibitors increased cell size and led to irregularly shaped nuclei. Heavy vacuolization was observed following HMA incubation, while apoptotic bodies and disintegration of the cell membrane were associated with VEN exposure. These characteristics hint at induction of apoptosis or other cell death mechanisms. As expected, annexin V/propidium iodide staining revealed significant apoptosis commitment following VEN in SEM and RS4;11 cells, while REH and NALM-6 are known to be intrinsically resistant (Figure 2B). The addition of HMA increased apoptosis induction in SEM, but not RS4;11 cells, with DEC being more effective than AZA. For SEM cells, simultaneous or DEC followed by VEN application offered a synergistic reduction of viable leukemia cells (Figure 2C,D).

**Figure S5:**
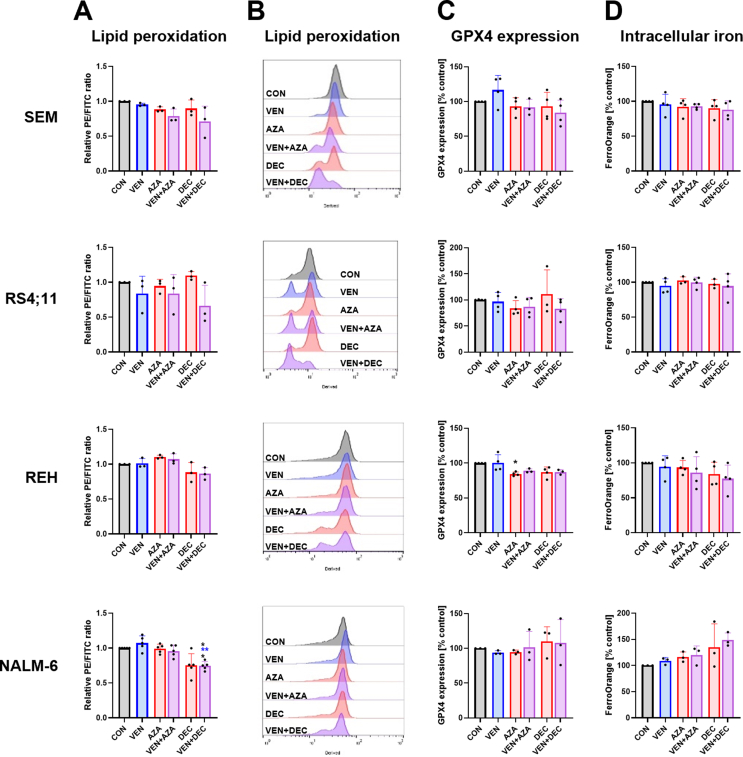
Influence of mono and combined application of VEN and HMA on ferroptosis induction. B-ALL cell lines SEM, RS4; 11, REH and NALM-6 were simultaneously incubated with inhibitors (VEN: SEM; 10 nM, RS4; 11, REH, NALM-6, all 2.5 nM; AZA, all 1 μM; DEC, all 100 nM) for 72 h **A** Flow cytometry-based assessment of lipid peroxidase activity. Each dot represents an individual biological replicate. Mean ± SD, 2-way ANOVA with post-hoc Tukey's multiple comparisons test. ∗ p < 0.05, ∗∗ p < 0.01 vs CON (black), VEN (blue) or HMA (red). **B** Representative images of histograms generated using lipid peroxidation flow cytometry. **C** GPX4 expression was measured by intracellular flow cytometry. Each dot represents an individual biological replicate. Mean ± SD, one-way ANOVA with post-hoc Sidák's multiple comparisons test (SEM, REH, NALM-6) or Kruskal–Wallis with post-hoc Dunn's multiple comparisons test (RS4; 11) based on Gaussian distribution. ∗ p < 0.05. **D** Intracellular iron was quantified using the FerroOrange probe. Each dot represents an individual biological replicate. Mean ± SD, one-way ANOVA with post-hoc Sidák's multiple comparisons test. ∗ p < 0.05.

To further investigate other cell death mechanisms, we analyzed lipid peroxidation, GPX4 expression and intracellular iron as markers of ferroptosis (Fig. S5). All four cell lines exhibited slightly increased lipid peroxidation, as indicated by reduced PE/FITC ratios, in response to combined VEN and HMA treatment, particularly following VEN + DEC application (Fig. S5A and B). In contrast, ferroptosis inhibitor GPX4 and intracellular iron levels were unaffected by all treatment regimens (Fig. S5C and D), indicating that combined VEN and HMA incubation does not influence ferroptosis induction.

### 3.3 VEN-resistant cell clusters exhibit impaired metabolism and energy supply

**Figure 3:**
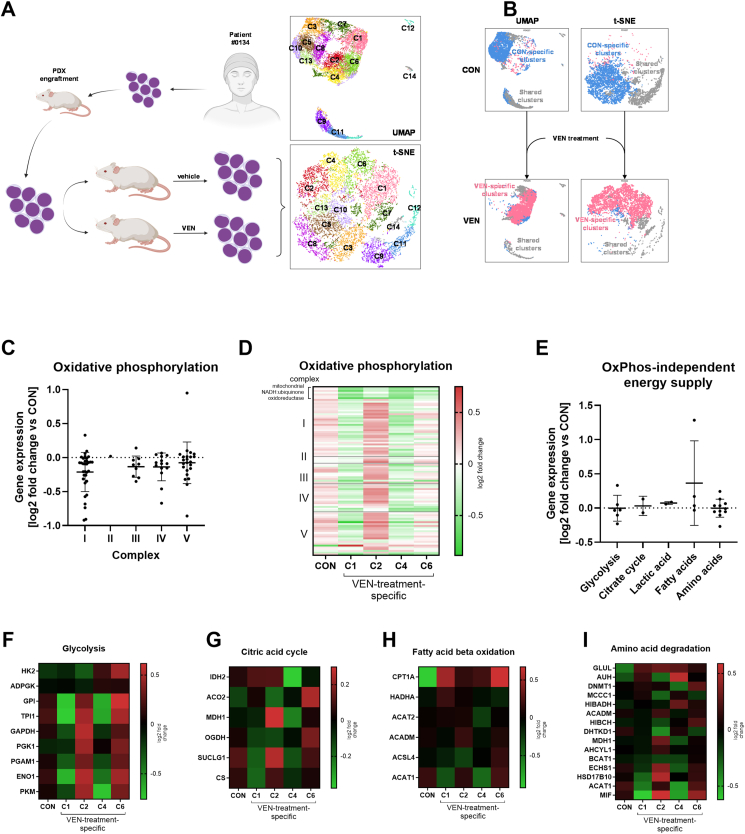
**Single cell RNAseq (scRNAseq)-based characterization of metabolic energy supply mechanisms. A** Patient 0134-derived B-ALL cells were serially transplanted in NSG mice and animals were treated with either vehicle or VEN (100 μg/kg p.o.) until peripheral blood blast frequency surpassed 30 %. Blasts were isolated from spleens and subjected to scRNAseq analysis. Data analysis revealed 14 distinct clusters as displayed in UMAP (top) and t-SNE plots (bottom). Created in BioRender. Richter, A. (2026) https://BioRender.com/t44z2yt. **B** Longitudinal visualization of clusters found exclusively in the control (CON, blue), VEN-treated animal (red) or both samples (Shared, grey) using UMAP (left) and t-SNE plots (right). **C** Gene expression analysis of genes involved in respiratory chain complexes I to V across all clusters in the VEN-treated animal compared to the control. Each dot represents an individual gene. **D** Heatmap demonstrating the gene expression of genes involved in oxidative phosphorylation in VEN-treatment-specific clusters 1, 2, 4 and 6 compared to the mean of the control-specific clusters. **E** Gene expression analysis of genes involved in oxidative phosphorylation-independent energy supply mechanisms across all clusters in the VEN-treated animal compared to the control. Each dot represents an individual gene. **F–I** Heatmaps demonstrating the gene expression of genes involved in oxidative phosphorylation-independent energy supply in VEN-treatment-specific clusters 1, 2, 4 and 6 compared to the mean of the control-specific clusters.

**Figure S6:**
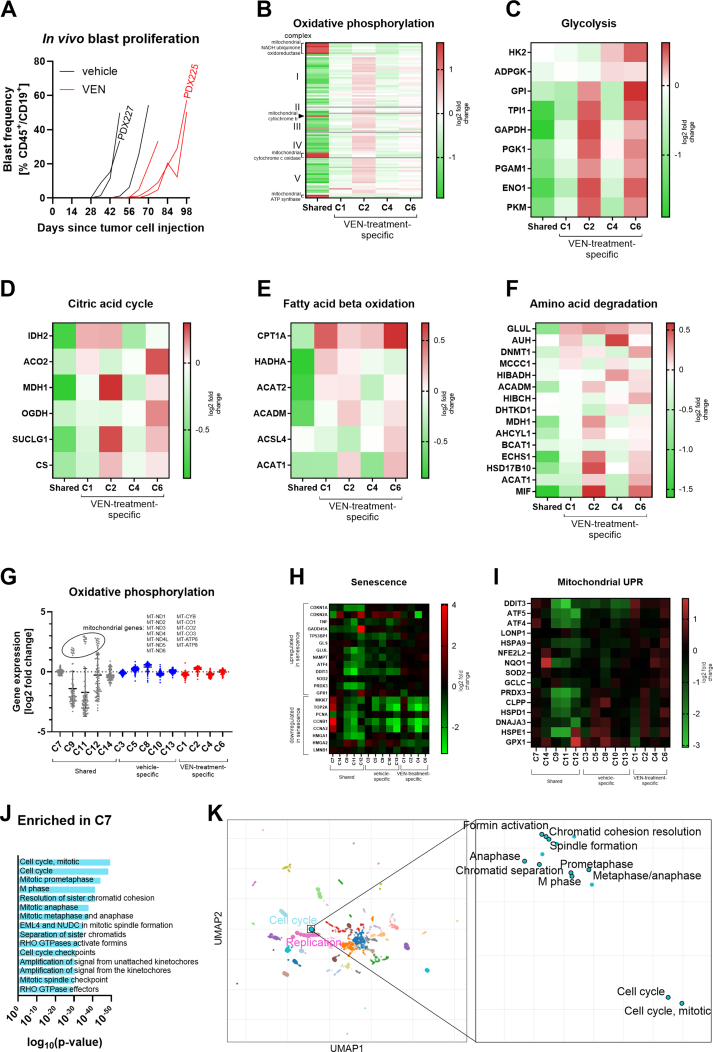
Single cell RNAseq-based characterization of metabolic energy supply mechanisms in cell clusters shared by control and VEN-treated animals. **A** Tumor cell proliferation in patient 0134-engrafted animals was measured by peripheral blood flow cytometry. Animals PDX227 (vehicle) and PDX225 (VEN) were used for subsequent single cell RNAseq analyses. **B–F** Heatmap demonstrating the gene expression of genes involved in oxidative phosphorylation (**B**), glycolysis (**C**), citric acid cycle (**D**), fatty acid beta oxidation (**E**) and amino acid degradation (**F**) in VEN-treatment-specific clusters 1, 2, 4 and 6 compared to the mean of the clusters shared between control and VEN-treated animals. **G** Gene expression analysis of genes involved in oxidative phosphorylation across all clusters. Each dot represents an individual gene. Mitochondrial genes upregulated in clusters 9, 11 and 12 are circled. **H,I** Heatmap demonstrating the gene expression of genes involved in senesecence (**H**) and mitochondrial UPR (**I**) across all clusters. **J** Enrichment analysis of genes upregulated in cluster 7. **K** UMAP plot representing pathway regulation in cluster 7 (left). The insert (right) is a magnification of processes involved in cell cycle regulation. The top-10 regulated pathways are indicated by black circles.

Apart from intrinsic apoptosis, mitochondrial function is further involved in cellular energy supply by oxidative phosphorylation. Also, several metabolic processes are involved in the cells’ energetic system. To investigate whether VEN impairs ATP production via the respiratory chain and/or other metabolic processes, we employed a B-ALL patient-derived xenograft model to mimic the multi-organ involvement of potential VEN resistance mechanisms. Primary cells were serially transplanted into NSG mice, and animals were continuously treated with either VEN or vehicle. As expected, VEN treatment initially prevented tumor cell proliferation, but resistance manifested around four weeks into drug application as indicated by rapidly increasing blast frequency in peripheral blood (Fig. S6A). Tumor cells were isolated upon profound leukemic manifestation in peripheral blood (Figure 3A).

Single cell RNA sequencing revealed cellular clusters specific for either control or VEN treatment. In addition, roughly 20 % of all cells shared a gene expression signature that was found in both, control and VEN-treated group, suggesting that these cells in clusters 7, 9, 11, 12 and 14 (“Shared”) were not greatly affected by VEN application and persisted in their initial state (Figure 3B). Their expression profiles were characterized by a low abundance of enzymes involved in oxidative phosphorylation, glycolysis, citrate cycle, fatty acid beta oxidation and amino acid degradation, indicating a subgroup of cells with weak metabolic activity and low ATP production that cannot be targeted by VEN therapy (Fig. S6B–F). However, mitochondrial genes involved in the respiratory chain complexes I, III, IV and V showed the opposite pattern in clusters 9, 11 and 12: the mitochondrial NADH:ubiquinone oxidoreductase (*MT–ND* genes), mitochondrial cytochrome b (*MT-CYB*), mitochondrial cytochrome c oxidase (*MT–CO* genes) and mitochondrial ATP synthase (*MT-ATP* genes) were all highly expressed in cells that did not respond to VEN treatment (Fig. S6B and G). This questions the previous conclusion that these cells are characterized by a reduced overall metabolism, and might hint at mitochondrial compensation. Further, the observed mito-nuclear dysregulation was not due to a senescent state or activated mitochondrial unfolded protein response in those cells (Fig. S6H and I). Instead, enrichment analyses of clusters 9 and 11 demonstrated mitochondrial upregulation of RNA processing, metabolism and degradation, while clusters 12 and 14 presented markers of disturbed iron homeostasis and oxygen exchange (Tables S4–8). These data indicate at subclones with severely impaired metabolism and activated rescue pathways. In contrast, cells in cluster 7 presented an intense upregulation of genes related to cell cycle progress, suggesting a therapy-refractory population that might be involved in resistance development (Fig. S6J and K, Table S9).

VEN induces apoptosis via BAX/BAK oligomerization and subsequent formation of pores in the outer mitochondrial membrane. As expected, mitochondrial processes are generally down-regulated in cells following VEN application, with decreased expression of genes encoding the enzymes of oxidative phosphorylation (Figure 3C). Complex I, especially the mitochondrial NADH:ubiquinone oxidoreductase, was affected the most. This was confirmed when examining the VEN-induced gene expression profiles in more detail: compared to control-specific cell clusters, the four cell clusters C1, C2, C4 and C6, which are exclusively found in the VEN-treated sample, demonstrated an intense reduction of the mitochondrial NADH:ubiquinone oxidoreductase (Figure 3D). Of note, other mitochondrial genes did not show a comparable pattern of downregulation. Instead, the response of the cell clusters to VEN was highly heterogeneous, with clusters 1, 4 and 6 exhibiting an overall decreased gene expression spanning the remaining enzymes of all five complexes of the respiratory chain. On the other hand, the entire oxidative phosphorylation cascade was upregulated in cluster 2, suggesting a mode of adaptive compensatory response to VEN treatment.

**Figure S7:**
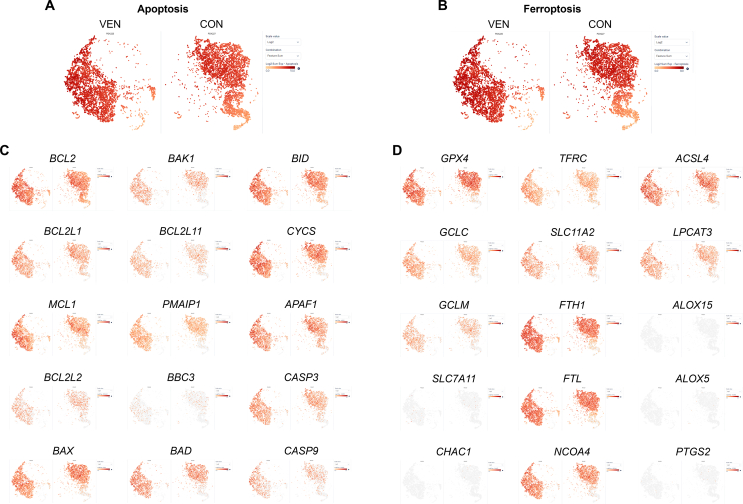
Expression of genes involved in apoptosis and ferroptosis in PDX samples treated with VEN or vehicle. t-SNE plots demonstrating log2-transformed UMI counts of the VEN-treated (left) and control sample (right). Lists of genes allocated to the pathways can be retrieved from tables S10 and S11. **A,B** Combined overall expression of genes responsible for intrinsic apoptosis commitment (A) and ferroptosis induction (B), respectively. **C,D** Single cell gene expression profiles of selected marker genes for apoptosis (C) and ferroptosis (D).

Investigating further energy supply mechanisms, no relevant changes in gene expression were observed for enzymes involved in carbon metabolism (glycolysis, citrate cycle, lactic acid fermentation), fatty acid beta oxidation or amino acid degradation, underlining the specificity of VEN as a mediator of mitochondrial function and oxidative phosphorylation (Figure 3E–I). Comparable to the genes involved in the respiratory chain, we once again found that the four VEN-specific clusters reacted highly heterogeneous, with clusters 2 and 6 demonstrating increased glycolysis, citrate cycle and amino acid degradation gene activation while clusters 1 and 4 showed reduced reads of those enzymes. Cross-evaluation of apoptosis and ferroptosis-indicating genes once again proved that transcriptional modulation of those processes does not play a central role in VEN efficacy (Fig. S7). While the analysis of the VEN-induced metabolic landscape at single cell resolution suggest far-reaching modulatory effects, it is limited to the gene expression level. Subsequent experiments are therefore required to determine the functional relevance of the transcriptomic changes.

### 3.4 VEN and HMA synergistically inhibit B-ALL cell metabolism

We next aimed to extend the VEN-evoked antimetabolic effect using HMA combinations, possibly adding glycolysis impairment to the VEN-induced reduction in respiratory chain gene expression. We therefore analyzed the effect on the cells’ overall metabolic activity, oxidative phosphorylation and glycolysis in response to inhibitor application.

**Figure 4:**
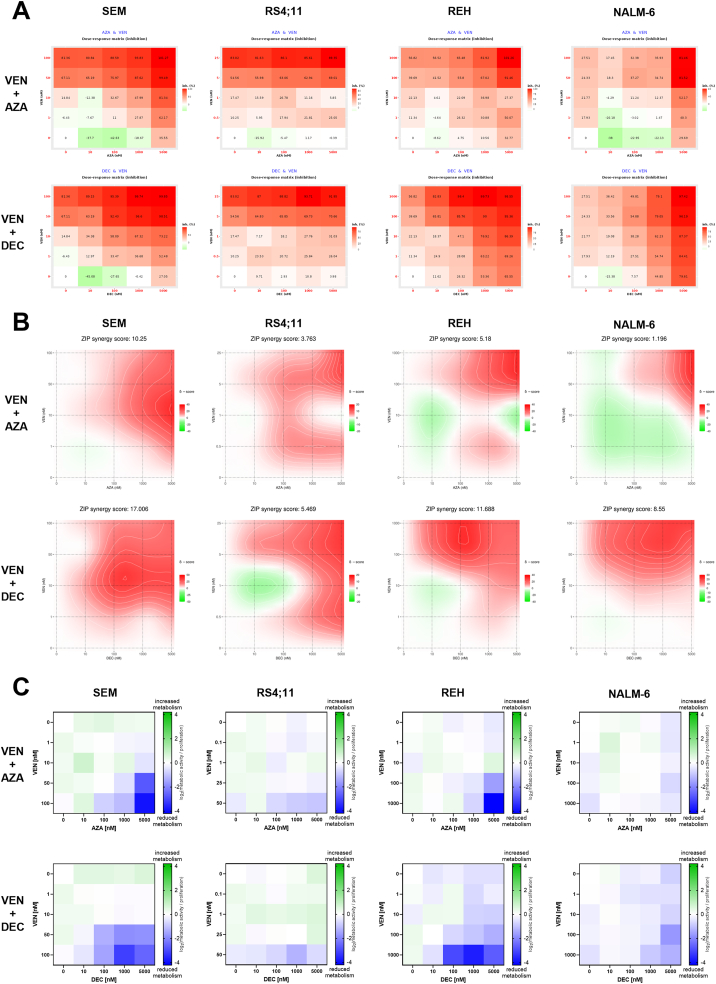
**Effects of combined VEN and HMA application on metabolic activity in B-ALL cell lines.** SEM, RS4;11, REH and NALM-6 cells were incubated with increasing concentrations of VEN and HMA for 72 h in three independent biological and technical replicates before WST-1 assay measurement. **A** Dose–response matrix of cross-titration experiments of combined VEN and HMA application in B-ALL cell lines using increasing concentrations of VEN, AZA (upper panel) and DEC (lower panel). Darker red shade indicates stronger anti-metabolic activity. ed color indicates high inhibitory capacity while while or green shades suggests therapeutic resistance. **B** Synergy maps of combined VEN + AZA (upper panel) and VEN + DEC application (lower panel) were generated using the SynergyFinder tool [19] based on the zero interaction potency (ZIP) model. Red color indicates synergistic potential while green color suggests antagonism. **C** Heatmaps representing the relative metabolic activity of cells treated with VEN + AZA (upper panel) or VEN + DEC (lower panel). The relative metabolic activity of each sample compared to the respective untreated control was divided through the relative amount of cells compared to the respective untreated control. Green color indicates increased metabolic activity compared to untreated controls while blue color suggests reduced relative metabolism.

**Figure S8:**
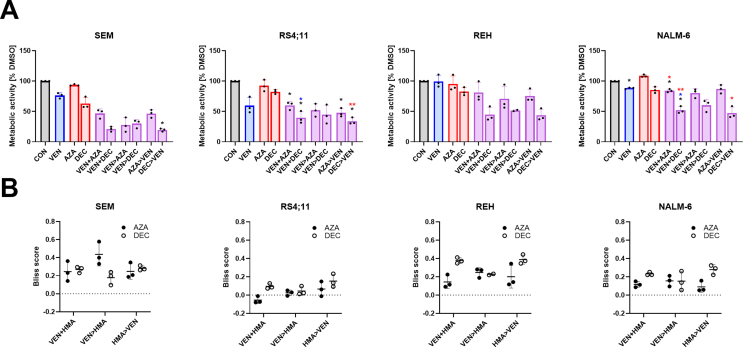
Effects of combined VEN and HMA application on metabolic activity in B-ALL cell lines. SEM, RS4; 11, REH and NALM-6 cells were incubated with VEN (SEM, 10 nM; RS4; 11, 2.5 nM; REH, 50 nM; NALM-6, 50 nM), AZA (all 1 μM) or DEC (all 100 nM) for 72 h. For simultaneous treatment (VEN + HMA), cells were seeded and incubated with both inhibitors for 72 h. For sequential application (VEN > HMA or HMA > VEN), cells were seeded and immediately incubated with the first drug. After 24 h, the second drug was added for a total cultivation period of 72 h. **A** WST-1 assay to assess metabolic activity following incubation with VEN (10 nM), HMA (AZA, 1 μM; DEC, 100 nM) or combined for 72 h. Each dot represents an individual biological replicate. Mean ± SD, repeated measures one-way ANOVA with post-hoc Tukey's multiple comparisons test or Friedman test with post-hoc Dunn's multiple comparisons test depending on Gaussian distribution of the raw data. ∗ p < 0.05, ∗∗ p < 0.01 vs CON (black), VEN (blue) or HMA (red). **B** Calculation of synergistic anti-metabolic activity according to the Bliss independence model based on the data presented in Figure S6A. Positive bliss scores indicate synergy while negative scores predict antagonism. Each dot represents an individual biological replicate.

Combined application resulted in significantly reduced cellular metabolism (Figure 4A, Fig. S8A), and the effect was highly synergistic for both, simultaneous and sequential treatment in all four cell lines (Fig. S8). Across all cell lines, DEC-containing combinations usually induced stronger anti-metabolic effects than AZA regimens, and ZIP and Bliss scores indicate higher synergy for the VEN/DEC application (Figure 4B, Fig. S8B). Again, and matching proliferation and apoptosis data, we discovered that AZA is more effective when applied after VEN. In contrast, combined VEN and DEC treatment should be conducted either simultaneously or beginning with DEC. To exclude that the observed anti-metabolic effects were compromised by reduced cellular proliferation, we calculated the relative metabolic activity of each sample (Figure 4C). The results demonstrate that combined VEN and HMA application indeed induces a severe impairment of the cells’ metabolic activity, while single agent incubation was less effective or even slightly increased individual cellular metabolism.

**Figure 5:**
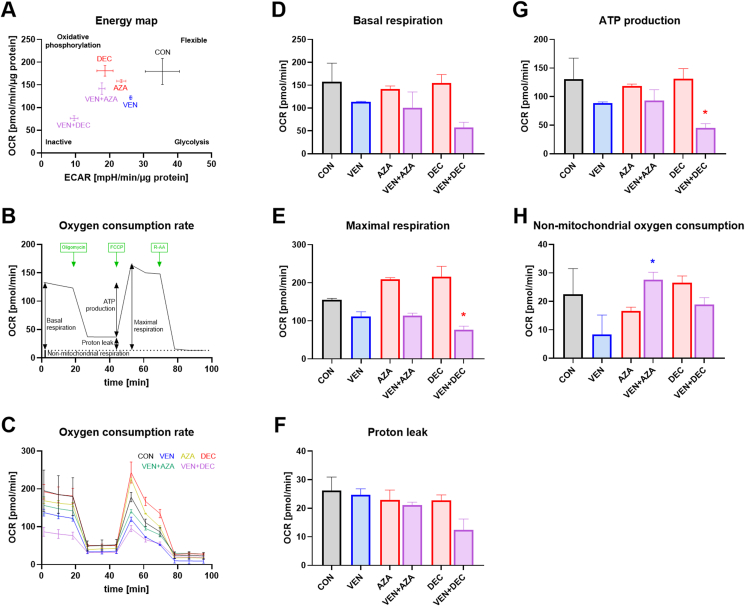
**Effects of combined VEN and HMA application on metabolic processes in B-ALL cell lines.** Cells were incubated with inhibitors (VEN: SEM, 10 nM; RS4;11, 2.5 nM; REH, 50 nM; NALM-6, 50 nM; AZA: all 1 μM; DEC: all 100 nM) for 72 h. **A** Energy map describing the changes of oxygen consumption rate (OCR) and extracellular acidification rate (ECAR) following 72 h control, VEN, HMA and combined application in SEM cells. N = 3; mean ± SEM. **B** Exemplary experiment protocol and readout parameters of the Seahorse Mito Stress test. **C** OCR analysis using the Seahorse Mito Stress test protocol of SEM cells treated for 72 h with control, VEN, HMA or both. N = 3, mean ± SD. **D-H** Determination of basal respiration (**D**), maximal respiration (**E**), proton leak (**F**), ATP production (**G**) and non-mitochondrial oxygen consumption (**H**) using the Seahorse Mito Stress test protocol of SEM cells treated for 72 h with control, VEN, HMA or both. N = 3, mean ± SD, Friedman test with post-hoc Dunn's multiple comparisons test. ∗p < 0.05 vs control (black), VEN (blue) or HMA (red).

Investigating metabolic processes in more detail, we determined the oxygen consumption rate (OCR) and extracellular acidification rate (ECAR) of SEM cells incubated with VEN, HMA or both (Figure 5). Again, combined VEN and DEC incubation induced greater changes than VEN/AZA and shifted the cells from a flexible metabolic plasticity state towards significantly decreased metabolic activity. This encompassed a decrease in oxidative phosphorylation activity (Figure 5A). Since ECAR in is strongly influenced by CO_2_-induced hyperactivity in glucose-containing media when using the Mito Stress Test, it cannot be directly interpreted as glycolytic flux. The energy map therefore only indicates an apparent decrease in ECAR after DEC, which most likely reflects reduced mitochondrial activity. However, a contribution from reduced glycolytic flux cannot be ruled out. AZA showed weaker effects on mitochondrial respiration, while VEN primarily reduced OCR. Combined VEN and AZA application did not greatly improve the effect of the single agents.

Further analyzing the different components of OCR to determine the exact effects evoked by the inhibitors (Figure 5B,C), we found a decreased basal and significantly reduced maximal respiration following combined VEN and DEC treatment (Figure 5D,E). Also, this combination was the only condition that reduced proton leakage as an indicator of ATP production-unrelated oxygen consumption (Figure 5F). This suggests that the cells efficiently use the available energy capacity to generate ATP. However, the overall ATP production rate in VEN/DEC-treated cells was significantly lower than in all other samples (Figure 5G), demonstrating extensive metabolic impairment and overall loss of mitochondrial respiration. As expected from the energy map (Figure 5A), single HMA application did not negatively interfere with any of the OCR components. Interestingly, both, AZA and DEC increased maximal respiration, demonstrating a high mitochondrial reserve capacity following HMA treatment. Non-mitochondrial oxygen consumption was decreased following VEN application, likely due to apoptosis-mediated downregulation of NADPH oxidases and subsequent decrease in reactive oxygen species production (Figure 5H).

## 4 Discussion

Combinatory approaches of targeted therapies are a key mechanism to increase therapeutic efficacy and reduce secondary resistance. Because of promising results in preclinical single compound application, outstanding clinical success in AML treatment and due to lack of data in B-ALL, we analyzed the anti-leukemic potential of combined VEN and HMA application in B-ALL cell lines and investigated the underlying modes of action. We identified HMA, especially DEC, as a potent combination partner for VEN and demonstrated synergistically impaired metabolic activity.

We found that the addition of HMA to VEN incubation increased anti-proliferative and pro-apoptotic effects even in cell lines intrinsically resistant to VEN. We were able to verify the hypothesis of Benito et al. claiming that KMT2A-rearranged B-ALL cells are more responsive than KMT2A-wild type cell lines [4], which also matches our previous data using patient-derived xenograft models [2]. Of note, these anti-leukemic effects were achieved using concentrations that did not interfere with the viability of healthy blood cells.

Investigating potential ways of synergistic action, we found that anti-proliferative effects and increased induction of apoptosis were rather additive but not synergistic. So far, further preclinical and clinical combinatory data in B-ALL are missing; but results in AML were comparable with modest synergistic potential of VEN and AZA application [29]. This matches the lack of influence on the BCL-2-mediated intrinsic apoptosis pathway protein expression: the addition of HMA did not alter any of the markers analyzed in all cell lines. Instead, different, albeit mild, changes were observed, with DEC increasing VEN-induced BCL-2 dephosphorylation in SEM cells, while this combination resulted in reduced MCL-1 expression in REH cells and upregulation of BAX in the cell line NALM-6. Overall, this demonstrates a heterogeneous picture between cell lines, suggesting that mediation of BCL-2 family proteins is not a general way to increase anti-leukemic activity in B-ALL cell lines.

Similar results were found for ferroptosis induction, an alternative cell death pathway. Matching preclinical AML data [30,31], we observed a mild increase in lipid peroxidation as a marker for ferroptosis following VEN, which was increased by DEC. Investigating ferroptosis in more detail, however, revealed that GPX4 and intracellular iron were unaffected by all treatment protocols, suggesting that cell death mechanisms in general, including ferroptosis, play a minor role in synergistic VEN and HMA anti-leukemic activity, which is also supported by concomitant single cell RNAseq experiments. Garcia-Gimenez et al. identified ferroptotic cell death as a mechanism of VEN action in B-ALL cells with hotspot mutations in the CREBBP acetyl-CoA binding site (p.R1446C) [32]. While NALM-6 also features a CREBBP mutation (p.S1687P), this residue is not involved in acetyltransferase activity, explaining the lack of ferroptosis induction in the CREBBP-mutant NALM-6 as well as CREBBP wild type cell lines SEM, RS4;11 and REH.

Overall, we detected stronger anti-leukemic effects with DEC compared to AZA, even using a ten times lower concentration. This also included global demethylation, likely due to the fact that DEC is solely incorporated into the DNA during replication while AZA is also integrated into RNA, accounting for less methylation-specific effects. Limited combinatory efficacy of VEN and AZA could be due to the simultaneous application: we found that proliferation, vitality and metabolic activity of leukemic cell lines was most effectively impaired when AZA was applied after VEN, while simultaneous inhibition or DEC first was better with the latter HMA. This once again suggests different modes of action between AZA and DEC. It is known that DEC is an inductor of senescence, while no such data is available for AZA [33,34]. Given this discrepancy, it appears possible that the DEC-VEN sequence acts as senescence-senolysis cascade. Navitoclax, a BCL-2/BCL-xL inhibitor, is a proven senolytic substance and structurally similar to VEN [35], suggesting that VEN might have senolytic properties as well.

As expected and matching existing literature [36,37], VEN alone reduced all parts of mitochondrial metabolism due to severe apoptosis induction based on pore formation in the outer mitochondrial membrane. The cells exhibited reduced oxidative phosphorylation and showed no signs of sufficient glycolytic compensation. However, several preclinical and clinical studies demonstrated the rise of VEN resistance and identified upregulation of genes involved in mitochondrial translation and overall oxidative phosphorylation as possible modes of resistance [[36], [37], [38]].

Although HMA acted differently in several analyzed mechanisms, both were able to decrease the metabolic activity of the cells and synergize with VEN, offering a promising strategy to overcome drug-induced metabolic adaptation as a mode of chemoresistance. Previous studies demonstrated that leukemic stem cells promote and use an altered niche metabolism to evade chemotherapy [39]. In line, Pollyea et al. have previously described that combined VEN and AZA application inhibits complex II of the electron transport chain, thus suppressing oxidative phosphorylation [40]. HMA AZA and DEC alone induced comparable effects on OCR and ECAR. Interestingly, the anti-leukemic potential of the VEN-DEC combination was once again higher compared to AZA, especially concerning ATP production and proton leakage. This combination markedly reduced oxidative phosphorylation and was associated with a limited metabolic compensatory response, consistent with a broad disruption of cellular energy supply pathways. This also underlines the findings of others who demonstrated that combined VEN and HMA treatment synergistically interfered with several pathways related to energy metabolism, including pyrimidine metabolism [41], ROS modulation [42] and mitochondrial apoptosis resistance [43].

Altogether, we were able to demonstrate that combined VEN and HMA application can act synergistically against ALL via alteration of metabolic processes. However, most of the herein presented data is limited by the in vitro character of the experiments and the small number of cell lines used. The results should be verified using primary ALL samples as well as cells collected from patients treated with VEN/HMA combination therapy, and mechanistic studies should be performed to achieve a deeper understanding of metabolic reprogramming evoked by this combination. Still, the application of VEN and HMA depicts a promising therapeutic option in ALL therapy.

## CRediT authorship contribution statement

**Patricia Maass:** Writing – review & editing, Visualization, Validation, Project administration, Methodology, Investigation, Formal analysis. **Sandra Lange:** Writing – review & editing, Validation, Supervision, Methodology, Investigation, Formal analysis. **Felix Wittig:** Writing – review & editing, Visualization, Validation, Supervision, Methodology, Investigation, Formal analysis, Data curation. **Nares Trakooljul:** Writing – review & editing, Validation, Supervision, Software, Project administration, Methodology, Formal analysis, Data curation. **Frieder Hadlich:** Writing – review & editing, Validation, Software, Methodology, Formal analysis, Data curation. **Anett Sekora:** Methodology, Investigation, Formal analysis. **Christian Schmidt:** Writing – original draft, Resources, Investigation, Formal analysis. **Hugo Murua Escobar:** Writing – review & editing, Resources, Project administration. **Klaus Wimmers:** Writing – review & editing, Supervision, Resources. **Burkhard Hinz:** Writing – review & editing, Supervision, Resources, Project administration. **Christian Junghanss:** Writing – review & editing, Supervision, Resources, Conceptualization. **Anna Richter:** Writing – review & editing, Writing – original draft, Visualization, Validation, Supervision, Resources, Project administration, Methodology, Investigation, Funding acquisition, Formal analysis, Data curation, Conceptualization.

## Funding

AR was a fellow of the Else Hirschberg program of the 10.13039/501100012688Rostock University Medical Center and the Professorinnenprogramm III of the 10.13039/501100012688University of Rostock. AR received funding from the FORUN program of the 10.13039/501100012688Rostock University Medical Center and the Lieselotte Beutel Stiftung. CS is a fellow of the Clinician Scientist program of the Comprehensive Cancer Center Mecklenburg-Vorpommern.

## Declaration of competing interest

The authors declare that they have no competing interests. AR was a fellow of the Else Hirschberg program of the Rostock University Medical Center and the Professorinnenprogramm III of the University of Rostock. AR received funding from the FORUN program of the Rostock University Medical Center and the Lieselotte Beutel Stiftung. CS is a member of the Clinician Scientist program of the Comprehensive Cancer Center Mecklenburg-Vorpommern.

## Data Availability

Data sets are available from the corresponding author upon reasonable request. Single cell raw data can be retrieved from the EBI's platform BioStudies (accession number E-MTAB-14805).
