# Supplementary material for: Venetoclax and hypomethylating agents synergize to increase cell death and metabolic remodeling in acute B-lymphoblastic leukemia cells
Source: Mol Metab. 2026 Jun 17;110:102402. doi: 10.1016/j.molmet.2026.102402 (PMC13326043; doi:10.1016/j.molmet.2026.102402)
Supplement: Multimedia component 1 [file mmc1.docx]

Table S1: Primers and probes used for gene expression analyses.

| Primer | Label | Sequence |
| --- | --- | --- |
| KMT2A-F | - | ACCCTGAGTGCCTTACCATG |
| KMT2A-R | - | TGGGGTGCCTTGTTTCTAGT |
| KMT2A probe | HEX | GAACCTCTTGCTCCACCCATCAAACCA |
| DNMT1-F | - | ACTGGCTTTGATGGAGGTGA |
| DNMT1-R | - | ACCGTGGTCTCGATCTTGTT |
| DNMT1 probe | FAM | AAGATTGTGGTGGAGTTCCTGCAGAGC |
| GAPDH-F | - | TCACCAGGGCTGCTTTTAAC |
| GAPDH-R | - | GGGTGGAATCATATTGGAACA |
| GAPDH probe | HEX | TGCCATCAATGACCCCTTCATTG |
| KMT2A::AFF1-F | - | CCGCCCAAGTATCCCTGTAA |
| KMT2A::AFF1-R | - | GCTCAGCTGTACTAGGCGTA |
| KMT2A::AFF1 probe | FAM | TGGCCGCCTCCTTTGACAGCA |
