# Supplementary material for: Venetoclax and hypomethylating agents synergize to increase cell death and metabolic remodeling in acute B-lymphoblastic leukemia cells
Source: Mol Metab. 2026 Jun 17;110:102402. doi: 10.1016/j.molmet.2026.102402 (PMC13326043; doi:10.1016/j.molmet.2026.102402)
Supplement: Multimedia component 2 [file mmc2.docx]

Table S2: Primers used for methylation-specific LINE-1 qPCR.

| Primer | Label | Sequence |
| --- | --- | --- |
| LINE-1_F_unmeth |  | TGTGTGTGAGTTGAAGTAGGGT |
| LINE-1_R_unmeth |  | ACCCAATTTTCCAAATACAACCATCA |
| LINE-1_F_meth |  | CGCGAGTCGAAGTAGGGC |
| LINE-1_R_meth |  | ACCCGATTTTCCAAATACGACCG |
