# Supplementary material for: Venetoclax and hypomethylating agents synergize to increase cell death and metabolic remodeling in acute B-lymphoblastic leukemia cells
Source: Mol Metab. 2026 Jun 17;110:102402. doi: 10.1016/j.molmet.2026.102402 (PMC13326043; doi:10.1016/j.molmet.2026.102402)
Supplement: Multimedia component 3 [file mmc3.docx]

Table S3: Antibodies used for intracellular flow cytometry.

| Target and fluorochrome | Brand | Cat-No |
| --- | --- | --- |
| BCL2-PE-Cy7 | Biolegend | 633512 |
| p-BCL2-Alexa Fluor 647 | Santa Cruz | sc-377554 AF647 |
| MCL-1-PE | Santa Cruz | sc-12756 PE |
| BCL-xL-PerCP-Cy5.5 | Santa Cruz | sc-8392 PerCPPC5 |
| BAX-PE | Santa Cruz | sc-7480 PE |
| GPX4 | Proteintech | CL488-30388 |
