# Supplementary material for: Venetoclax and hypomethylating agents synergize to increase cell death and metabolic remodeling in acute B-lymphoblastic leukemia cells
Source: Mol Metab. 2026 Jun 17;110:102402. doi: 10.1016/j.molmet.2026.102402 (PMC13326043; doi:10.1016/j.molmet.2026.102402)
Supplement: Multimedia component 10 [file mmc10.docx]

Table S10: Genes included in the Apoptosis feature list for single cell RNA sequencing.

| AKT1 | CASP10 | FAS | JUN | SCAF11 |
| --- | --- | --- | --- | --- |
| APAF1 | CASP2 | FASLG | LTA | TNF |
| BAD | CASP3 | GZMB | MAP2K4 | TNFRSF10B |
| BAK1 | CASP4 | HELLS | MAP3K1 | TNFRSF1A |
| BAX | CASP6 | HRK | MAPK10 | TNFRSF21 |
| BBC3 | CASP7 | IGF1 | MCL1 | TNFRSF25 |
| BCL2 | CASP8 | IGF1R | MDM2 | TNFSF10 |
| BCL2L1 | CASP9 | IGF2 | MYC | TP53 |
| BCL2L11 | CDKN2A | IKBKB | NFKB1 | TP63 |
| BCL2L2 | CFLAR | IKBKG | NFKBIA | TP73 |
| BID | CHUK | IRF1 | NFKBIB | TRADD |
| BIRC2 | CRADD | IRF2 | NFKBIE | TRAF1 |
| BIRC3 | CYCS | IRF3 | PIK3R1 | TRAF2 |
| BIRC5 | DFFA | IRF4 | PMAIP1 | TRAF3 |
| BNIP3L | DFFB | IRF5 | PRF1 | XIAP |
| BOK | DIABLO | IRF6 | RELA |  |
| CASP1 | FADD | IRF7 | RIPK1 |  |
