# Supplementary material for: Venetoclax and hypomethylating agents synergize to increase cell death and metabolic remodeling in acute B-lymphoblastic leukemia cells
Source: Mol Metab. 2026 Jun 17;110:102402. doi: 10.1016/j.molmet.2026.102402 (PMC13326043; doi:10.1016/j.molmet.2026.102402)
Supplement: Multimedia component 11 [file mmc11.docx]

Table S11: Genes included in the Ferroptosis feature list for single cell RNA sequencing.

| GPX4 | CHMP5 | GCLM | PCBP2 | SLC7A11 |
| --- | --- | --- | --- | --- |
| ACSL1 | CHMP6 | GSS | PHKG2 | STEAP3 |
| ACSL3 | CISD1 | HMGCR | POR | TF |
| ACSL4 | COQ2 | HMOX1 | PRNP | TFRC |
| ACSL5 | CP | HSPB1 | SAT1 | TP53 |
| ACSL6 | CTH | IREB2 | SAT2 | TXNRD1 |
| AIFM2 | CYBB | LPCAT3 | SLC11A2 | VDAC2 |
| AKR1C3 | DPP4 | MAP1LC3A | SLC1A5 | VDAC3 |
| ALOX15 | FDFT1 | MAP1LC3B | SLC38A1 | CHAC1 |
| ATG5 | FTH1 | NCOA4 | SLC39A14 | PTGS2 |
| ATG7 | FTL | NOX1 | SLC39A8 | ALOX5 |
| BACH1 | GCH1 | NOX4 | SLC3A2 |  |
| CBS | GCLC | PCBP1 | SLC40A1 |  |
